# On the mechanism of action of gated molecular baskets: The synchronicity of the revolving motion of gates and in/out trafficking of guests

**DOI:** 10.3762/bjoc.8.9

**Published:** 2012-01-16

**Authors:** Keith Hermann, Stephen Rieth, Hashem A Taha, Bao-Yu Wang, Christopher M Hadad, Jovica D Badjić

**Affiliations:** 1 Department of Chemistry, The Ohio State University, 100 West 18th Avenue, Columbus OH, 43210, USA

**Keywords:** dynamic NMR, host–guest chemistry, linear free-energy relationships, molecular encapsulation, recognition kinetics

## Abstract

We used dynamic ^1^H NMR spectroscopic methods to examine the kinetics and thermodynamics of CH_3_CCl_3_ (**2**) entering and leaving the gated molecular basket **1**. We found that the encapsulation is first-order in basket **1** and guest **2**, while the decomplexation is zeroth-order in the guest. Importantly, the interchange mechanism in which a molecule of CH_3_CCl_3_ directly displaces the entrapped CH_3_CCl_3_ was not observed. Furthermore, the examination of the additivity of free energies characterizing the encapsulation process led to us to deduce that the revolving motion of the gates and in/out trafficking of guests is synchronized, yet still a function of the affinity of the guest for occupying the basket: Specifically, the greater the affinity of the guest for occupying the basket, the less effective the gates are in “sweeping” the guest as the gates undergo their revolving motion.

## Introduction

Covalent and self-assembled molecules with a natural cavity, i.e., molecular capsules [1–2], employ several mechanisms to trap and release guests capable of residing in their inner space [3–5]. The so-called “slippage” scenario [6], in which a guest makes its way to and from the host by forcing the expansion of its aperture [7], appears frequently. The “gating” scenario [8], on the other hand, includes a conformational change in the host to create an opening that is large enough for a guest to “squeeze” its way in or out of the host. In the case of self-assembled hosts, however, the slippage, gating and possible partial or full disassembly of the capsule constitute mechanistic alternatives for the exchange of guests [4]. In the last decade, we [9–14] and others [7–8,15–18] have studied gated molecular encapsulation in artificial and natural systems [19].

**Figure 1:**
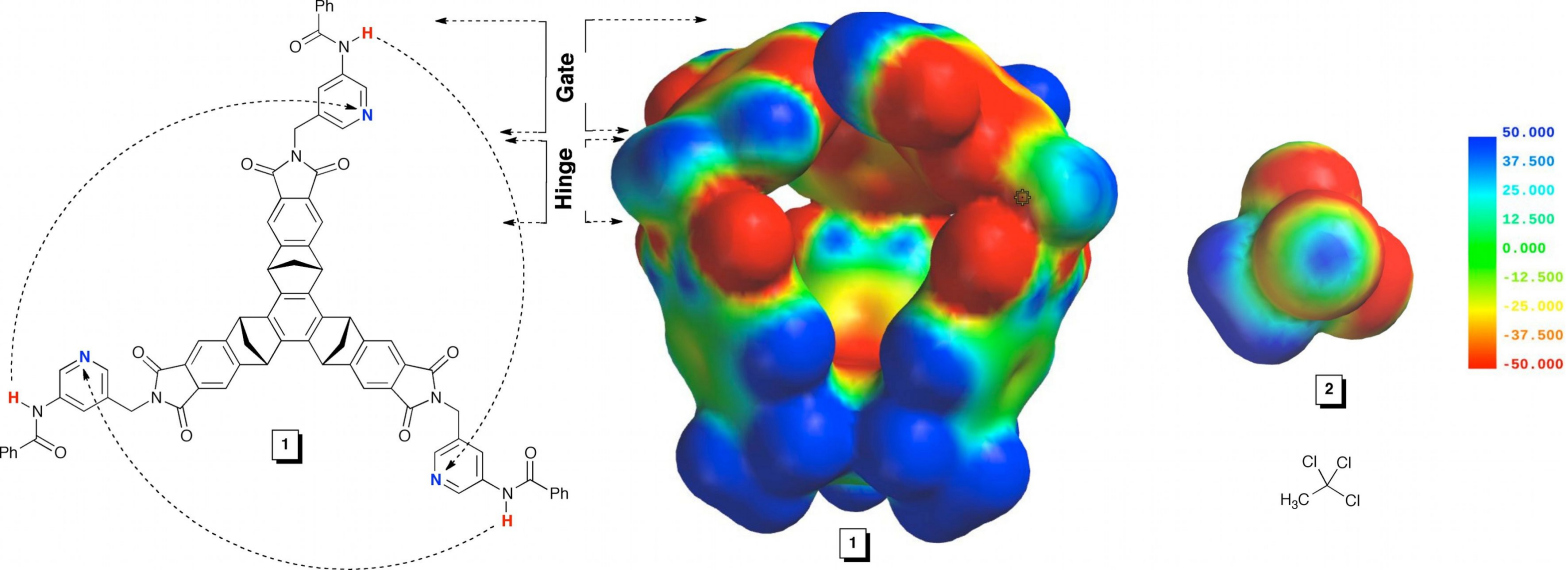
Chemical structure of gated molecular basket **1** and 1,1,1-trichloroethane (**2**). Electrostatic potential surfaces of basket **1** and guest **2** were computed with Spartan (AM1) [20].

In particular, we designed gated molecular baskets (Figure 1) and employed both experimental and theoretical methods to gain an understanding of their mechanism of action [4]. These dynamic hosts comprise a semirigid platform with three aromatic gates appended to its rim through CH_2_ “hinges” (Figure 1). The gates were set to interact by hydrogen bonding to control the opening and closing of the basket and thereby the rate by which a guest enters or departs the cavity of the basket [12–14]. Indeed, the action mechanism of the basket has been addressed [14], yet the exact role of the gates in the process of the in/out guest exchange necessitates additional scrutiny. In particular, a careful inspection of the additivity of free energies [21] pertaining to the constrictive Δ*G*^‡^_in/out_ and intrinsic Δ*G*° binding energies of the guests [11] as well as the racemization of the basket Δ*G*^‡^_rac_ (i.e., opening and closing, see below in Figure 6) reveals a systematic disparity (Δ*G*° + Δ*G*^‡^_rac_ + Δ*G*^‡^_sterics_ ≠ Δ*G*^‡^_out_, see below in Figure 7). In order to address this conundrum, we have employed methods of experimental (dynamic NMR) and computational chemistry (steered molecular dynamics, SMD) to inspect the relationship between the gates revolving at the rim of the host and the in/out exchange of guests. The results of our study suggest that for guests with a greater propensity to occupy the interior of the basket (i.e., more negative Δ*G*°) the process of gating is poorly synchronized with the guest exchange. The gates undergo a revolving motion to sweep the space but are concurrently less effective in enforcing the ejection of the guest from the cavity. Moreover, the results of dynamic ^1^H NMR measurements of CH_3_CCl_3_ (**2**) entering and departing basket **1** (Figure 1) suggest the absence of an interchange mechanism [22] in which a molecule of CH_3_CCl_3_ directly displaces another CH_3_CCl_3_ residing in the interior of the gated basket.

## Results and Discussion

### The encapsulation stoichiometry and the intrinsic binding (Δ*G*°)

**Figure 2:**
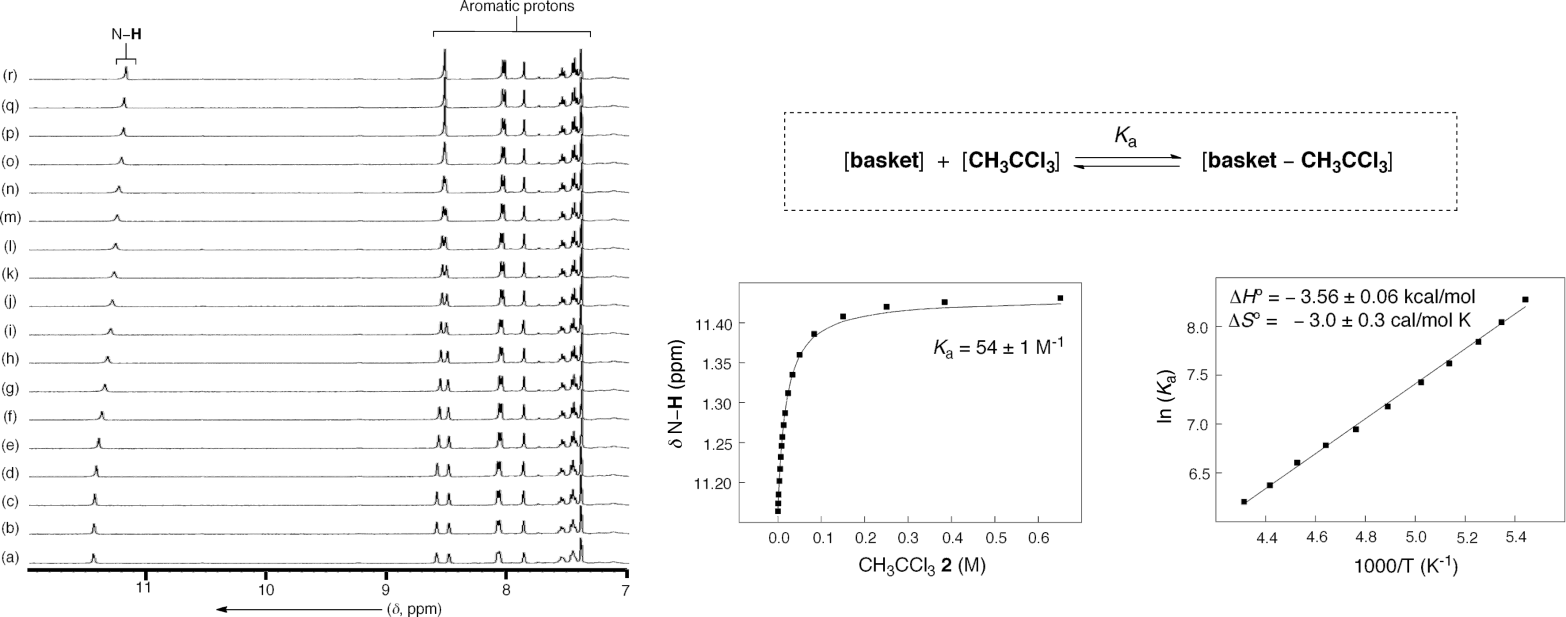
(Left): ^1^H NMR spectra (400 MHZ, CD_2_Cl_2_) of **1** (0.67 mM) obtained upon incremental addition of 1,1,1-trichloroethane (**2**) (0.00–0.65 M) at 298.0 K. (Middle): Nonlinear least-squares fitting (SigmaPlot) of the N–**H** chemical shift of **1** as a function of the concentration of **2** gave *K*_a_ = 54 ± 1 M^−1^ at 298.0 K [23]. (Right): The van't Hoff plot was generated from variable temperature ^1^H NMR measurements (400 MHz, 180–250 K) of **1** (0.67 mM) containing CH_3_CCl_3_ (1.07 mM).

In an earlier study [13], we reported on the tendency of basket **1** to trap CH_3_CCl_3_ (**2**) as a guest, and we hereby elaborate on the equilibrium thermodynamics of the recognition event (Figure 2). The incremental addition of **2** to a CD_2_Cl_2_ solution of **1** (0.67 mM, 298.0 K) caused considerable ^1^H NMR chemical shifts of the resonances corresponding to the presence of the basket (Figure 2). At 298.0 K, the formation and degradation of [basket–CH_3_CCl_3_] complex was sufficiently fast on the “NMR time scale”: The nonlinear least-squares fitting of the binding isotherm to a 1:1 binding model provided *K*_a_ = 54 ± 1 M^−1^ (*R*^2^ = 0.998, Figure 2) [23].

Indeed, the results of a variable temperature ^1^H NMR study (400 MHz, CD_2_Cl_2_) of **1** (0.67 mM) containing CH_3_CCl_3_ (**2**) (1.07 mM) was in line with the formation of the 1:1 complex; note that extrapolation of the fitted line gives *K*_a_ of 86 ± 16 M^−1^ at 298.0 K, which is akin to the value obtained in the titration experiment. Furthermore, the van't Hoff analysis of the ^1^H NMR data revealed that the encapsulation is also driven by enthalpy (Δ*H*° = −3.56 ± 0.06 kcal/mol, Figure 2). Indeed, the computed electrostatic potential surface (AM1, Spartan) [20] of guest **2** is complementary to the one corresponding to the concave interior of **1** (Figure 1). Furthermore, compound **2** (93 Å^3^, Spartan) occupies 42% of the inner space of **1** (221 ± 9 Å^3^) [11], which is close to the packing coefficient of liquids and thereby a good indicator of a stable assembly [24].

### The rate law characterizing guest exchange and the constrictive binding (Δ*G*^‡^_in/out_)

**Figure 3:**
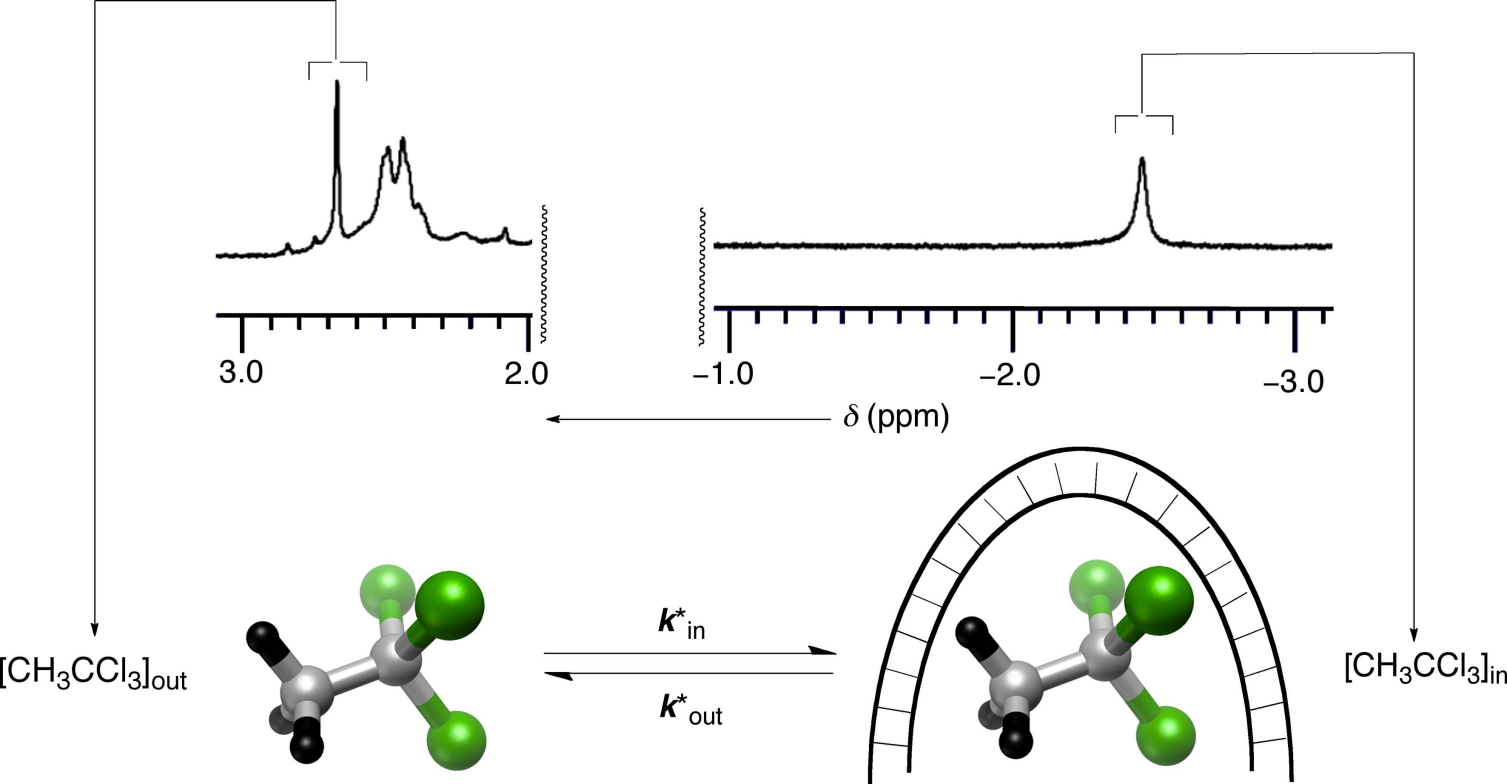
Chemically equivalent CH_3_ protons (black) in 1,1,1-trichloroethane (**2**) alter their magnetic environment from 2.70 ppm in bulk solvent to −2.45 ppm inside the basket.

We performed ^1^H,^1^H-EXSY [25] and selective inversion-transfer [26–27] NMR measurements (400 MHz, CD_2_Cl_2_) to examine the rate laws characterizing the trafficking of CH_3_CCl_3_ (**2**) to and from basket **1**. At concentrations of CH_3_CCl_3_ as a guest comparable to those of host **1**, the EXSY measurements (250.0 ± 0.1 K) allowed us to extract (MNova software) the magnetization rate coefficients k*_in_ and k*_out_ (Figure 3).

At higher concentrations of CH_3_CCl_3_ with respect to host **1**, however, we noticed an intense *T*_1_ noise coinciding with the [CH_3_CCl_3_]_out_ signal, thus preventing the accurate determination of the volume of the corresponding cross peak. Accordingly, we had to turn to selective inversion-transfer NMR measurements to obtain the values of k*_in_ and k*_out_. The exchange rate constants k*_in_ and k*_out_ (characterizing the longitudinal magnetization of the hydrogen nuclei in C**H**_3_CCl_3_ altering the chemical/magnetic environment) are by the nature of the experiment pseudo-first-order in character (see below) [25–26].

On the basis of the reaction stoichiometry (Figure 2), we initially made the assumption that the entrapment is first-order in both [basket] and [CH_3_CCl_3_]. Accordingly, the rate of the forward reaction is given as:

[1]
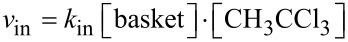


As per the earlier discussion, the pseudo-first-order constant k*_in_ describes the longitudinal magnetization of the hydrogen nuclei in C**H**_3_CCl_3_ transferring from the bulk solvent (δ = 2.70 ppm, Figure 3) to the interior of **1** (δ = −2.45 ppm, Figure 3).

Correspondingly, the rate of the forward reaction (entrapment) can be formulated as:

[2]
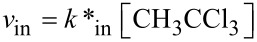


From Equation 1 and Equation 2, we furthermore derive:

[3]
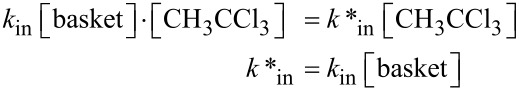


**Figure 4:**
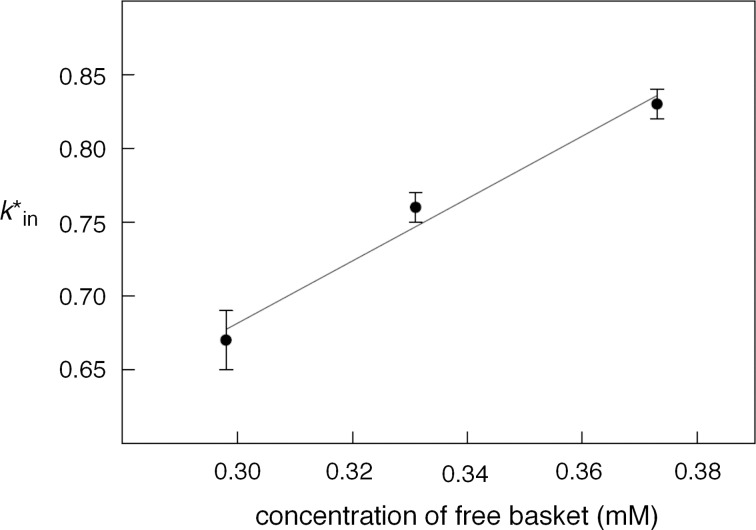
Nonlinear least-squares fitting (SigmaPlot) of magnetization rate constants k*_in_ (2-D EXSY, 250.0 ± 0.1 K) as a function of the concentration of the free basket to a linear function gives a slope of *k*_in_ = 2.1 ± 0.3 × 10^3^ M^−1^·s^−1^.

If the proposed model is valid, then the experimentally determined k*_in_ will be linearly proportional to the concentration of free basket **1**. Indeed, when the value of k*_in_ is plotted against the concentration of free basket **1**, there is an apparent linear dependence, with the slope of the fitted curve *k*_in_ = 2.1 ± 0.3 × 10^3^ M^−1^·s^−1^ (at 250 ± 0.1 K, Figure 4). Using Equation 4, we derive Equation 5, which upon insertion into Equation 3 gives Equation 6:

[4]
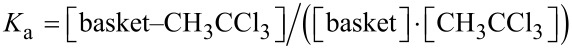


[5]
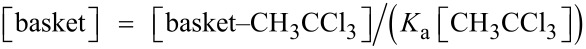


[6]
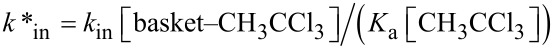


This particular dependence suggests that k*_in_ should be directly proportional to the concentration of the host–guest complex, [basket–CH_3_CCl_3_], but inversely proportional to the concentration of CH_3_CCl_3_. At higher concentrations of CH_3_CCl_3_, however, there should be a negligible variation in the concentration of basket–CH_3_CCl_3_ and the magnetization rate coefficient k*_in_ becomes inversely proportional to the concentration of CH_3_CCl_3_.

**Figure 5:**
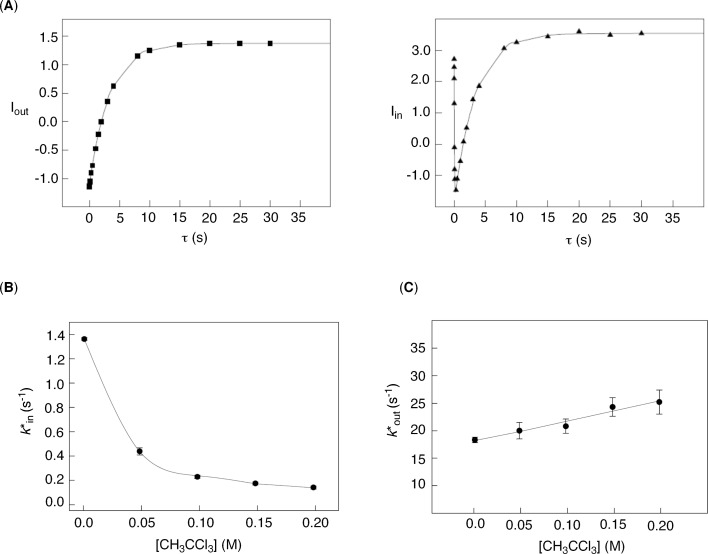
(A): Nonlinear least-squares fitting of ^1^H NMR signal intensities (I_in/out_) of [CH_3_CCl_3_]_in/out_ as function of the time variable τ (250.0 ± 0.1 K) was completed with the assistance of the Bloch–McConnell equations [27–28] describing the relaxation of hydrogen nuclei in two different environments; in this particular experiment [basket]_0_ = 1.65 mM and [CH_3_CCl_3_]_0_ = 50.0 mM. Magnetization transfer rate coefficients k*_in_ (B) and k*_out_ (C) were further obtained [27–28] from selective inversion-transfer measurements and plotted as a function of the concentration of free CH_3_CCl_3_.

In accordance with this theoretical model, we completed a series of selective inversion-transfer [27] NMR measurements of **1** (1.65 mM) and CH_3_CCl_3_ (16–200 mM) in CD_2_Cl_2_ at 250.0 ± 0.1 K (Figure 5). In the experiment, the proton resonance corresponding to [CH_3_CCl_3_]_out_ was selectively inverted, resulting in the perturbation of the longitudinal relaxation of both [C**H**_3_CCl_3_]_out_ and [C**H**_3_CCl_3_]_in_ due to chemical exchange over the course of variable delay time τ (180° x (selective) – τ – 90° x (nonselective) – τ_d_). Upon the integration of both signals (I_in_ and I_out_), we subjected the data to nonlinear least-squares fitting of I_in/out_ versus τ using the proposed solutions of the McConnell equations [27] describing the relaxation of the hydrogen nucleus residing in two environments (Figure 5A). For the fitting, the longitudinal relaxation rate (1/*T*_1_) of hydrogen nuclei in CH_3_CCl_3_ was determined separately by using a classical selective inversion-recovery NMR pulse sequence. When the experimental k*_in_ was plotted against the equilibrium concentration of CH_3_CCl_3_, there indeed appeared a hyperbolic dependence (Figure 5B) in agreement with Equation 6 (k*_in_ 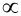 1/[CH_3_CCl_3_]). The fitting of the data to Equation 6 was inaccurate as only a few experimental points characterize the dependence (Figure 5B), although computing *k*_in_ from each data point would give a value of this coefficient (~2 × 10^3^ M^−1^·s^−1^) similar to that determined in the EXSY experiment (Figure 4). In accordance with the 2-D EXSY and selective inversion-transfer results, we conclude that the entrapment is first-order in both basket **1** and guest **2**.

On the basis of the reaction stoichiometry (Figure 2), the rate law for **2** leaving the encapsulation complex can be described as:

[7]
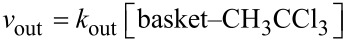


Alternatively, the rate of the same process expressed through the NMR magnetization transfer rate coefficient k*_out_ is:

[8]
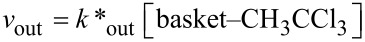


As in the case above, the manipulation of Equation 7 and Equation 8 gives Equation 9:

[9]
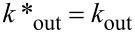


In accordance with this theoretical model, we increased the concentration of guest **2** (16–200 mM) with respect to **1** (1.65 mM) and measured k*_out_ using the selective inversion-transfer NMR pulse sequence. Markedly, there was essentially no interdependence between k*_out_ (21 ± 3 s^−1^) and the concentration of guest **2** (Figure 5C); the curve indeed shows a small slope, but the intercept of 18.1 suggests that this is likely an artifact. 2-D EXSY measurements would give a rate coefficient k*_out_ = 10 ± 0.1 s^−1^, which was also found to be independent of the external concentration of the basket/guest (Figure 4). The departure of CH_3_CCl_3_ from its complexed form [basket–CH_3_CCl_3_], therefore, follows a dissociative mechanism [4]. Notably, a molecule of solvent CD_2_Cl_2_ and not another CH_3_CCl_3_ (interchange mechanism) displaces the encapsulated guest. In fact, the inspection of CPK models as well as molecular dynamics studies (see below) revealed that the departure of CH_3_CCl_3_ (93 Å^3^) demands (a) “opening” of at least two gates, (b) disruption of internal N–H^…^N hydrogen bonds, and (c) distortion of the framework of the basket. We further reason that in the case of a direct exchange of two CH_3_CCl_3_ molecules, the departure of CH_3_CCl_3_ would create an empty host, and therefore vacuum, before another guest of the same kind can take its place. Note that two large compounds (overall ~186 Å^3^) cannot simultaneously occupy the interior of **1** (~220 Å^3^).

### Computational examination of the in/out trafficking

**Figure 6:**
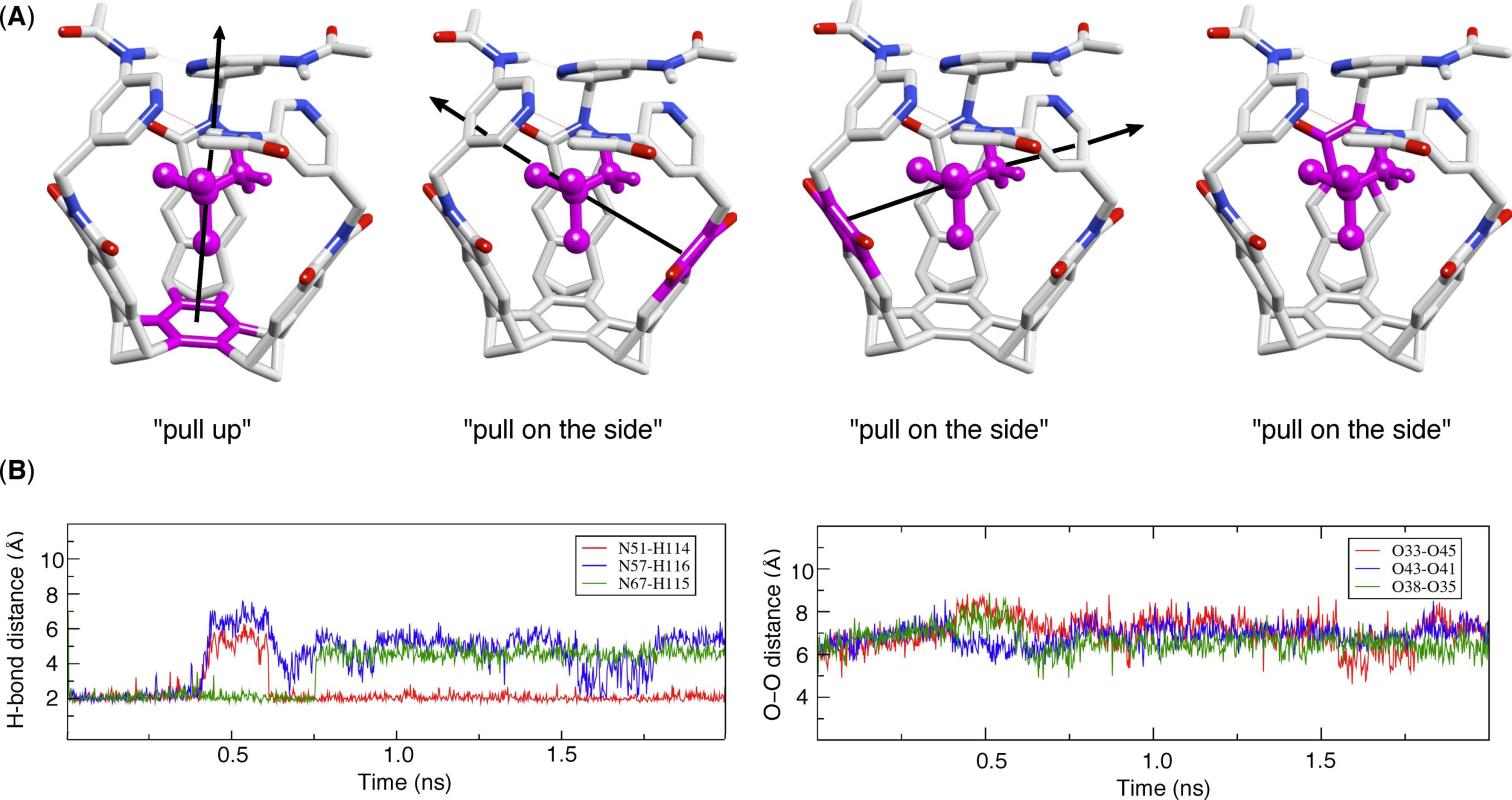
(A) Four different trajectories were used for examining the departure of CH_3_CCl_3_ guest from basket **1** with steered molecular dynamics. (B) The variation in N–**H**^…^**N and** –C=**O**^…^**O**=C– distances during SMD simulation with CH_3_CCl_3_ being pulled on the side.

To gain mechanistic insight into the departure of CH_3_CCl_3_ (**2**) from the interior of basket **1**, we completed a series of steered molecular dynamics (SMD) simulations using the AMBER 10.0 suite of programs [29–32]. Without applying any external force on the entrapped CH_3_CCl_3_, we first found that this guest would, within 10 ns, adopt many positions inside host **1**, although the one depicted in Figure 6A is obtained after 1 ns (Supporting Information File 1). The N–**H**^…^**N** hydrogen bond contacts along the top of the basket were also monitored throughout the 10 ns simulation. Importantly, the distance between each pair of amide-hydrogen and pyridine-nitrogen atoms was found to be invariant (~2 Å, see Supporting Information File 1).

In addition, the width of each side aperture (the span between adjacent carbonyl oxygen atoms) also remained constant at ~6.3 Å throughout the simulation (Figure S3, see Supporting Information File 1). We then selected multiple trajectories for “pulling” the guest from the host (Figure 6A). Markedly, the departure of CH_3_CCl_3_ necessitated the cleavage of at least two intramolecular N–**H**^…^N hydrogen bonds in **1** (Figure 6B) with a simultaneous expansion of the host (Figure 6B). That is to say, the “slippage” of CH_3_CCl_3_ (with gates in the “closed” position) does not appear to be a viable mechanistic scenario. Note that our simulation did not include solvent molecules (CD_2_Cl_2_) displacing the entrapped CH_3_CCl_3_, as suggested by the kinetic study. The substitution of the guest by the solvent should perhaps cause an even greater distortion of the framework of the basket.

### The revolving of the gates and the racemization of basket 1

**Figure 7:**
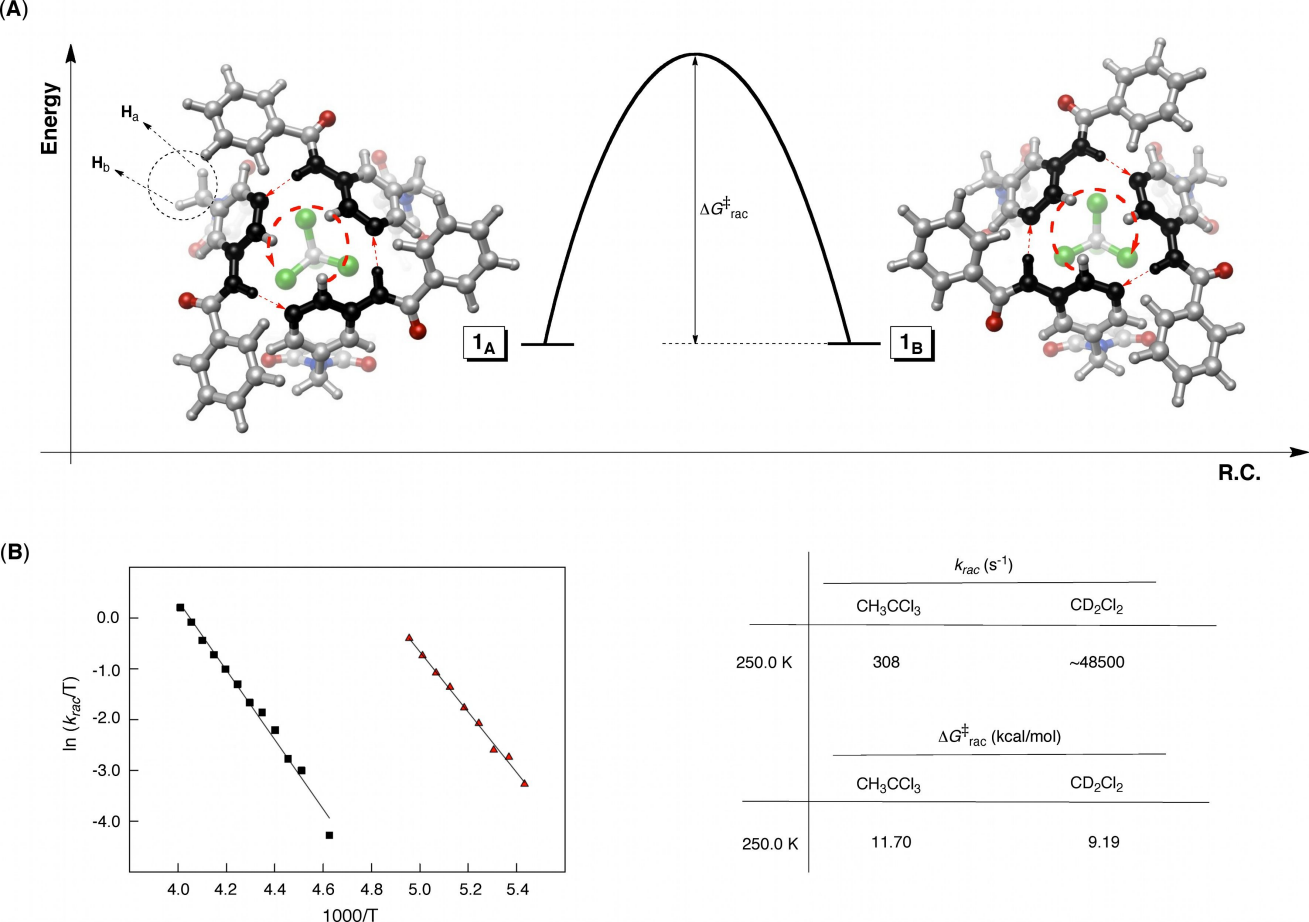
(A) The interconversion of conformational enantiomers **1**_A_ and **1**_B_, having anticlockwise and clockwise senses in the orientation of the intramolecular N–H^…^N hydrogen bonds, contributes to the process of racemization, i.e., the opening and closing of the basket [13]. (B) Eyring plots describing the linear relationship between ln(*k*_rac_/T) and temperature for basket **1** containing CH_3_CCl_3_ (black squares) and solvent CD_2_Cl_2_ (red triangles). The plots were generated from the results of the line-shape analysis (WinDNMR-Pro) of ^1^H NMR **H**_a/b_ signals of **1** (1.65 mM, CD_2_Cl_2_) at variable temperatures.

The aromatic gates in basket **1** interact through hydrogen bonding, as exemplified by a large downfield shift of the signal corresponding to (O=C)N−**H** protons (δ = 11.6 ppm at 298.0 K, Figure 2) [13]. In addition, the aromatic gates are dynamic, each one revolving about its axis to give rise to two enantiomeric conformers **1_A_** and **1_B_** (Figure 7A). The interconversion kinetics of the **1_A_**_/_**_B_** racemization can be followed by dynamic NMR spectroscopy in which a singlet corresponding to **H**_a_/**H**_b_ nuclei at high temperatures is seen to split into two doublets at low temperatures. In particular, the revolving rate of the gates is temperature dependent, thereby governing the lifetime of **H**_a_ or **H**_b_ nuclei, each residing in a particular chemical environment (τ = 1/*k*_rac_); the hydrogen nuclei are observed as separate resonances when τ >> 1/Δν(H_a/b_) [33]. Accordingly, we performed the classical line-shape analysis of **H**_a_/**H**_b_ resonances (WinDNMR-Pro software) to obtain the rate constants (*k*_rac_) and corresponding activation energies Δ*G*^‡^_rac_ characterizing the racemization of basket **1** (Figure 7B). Evidently, the rate at which the aromatic gates in **1** revolve is a function of the compound occupying the inner space: With CH_3_CCl_3_ the gates are less dynamic than with CD_2_Cl_2_ occupying the cavity (Figure 7B).

### On the action mechanism of the basket

**Figure 8:**
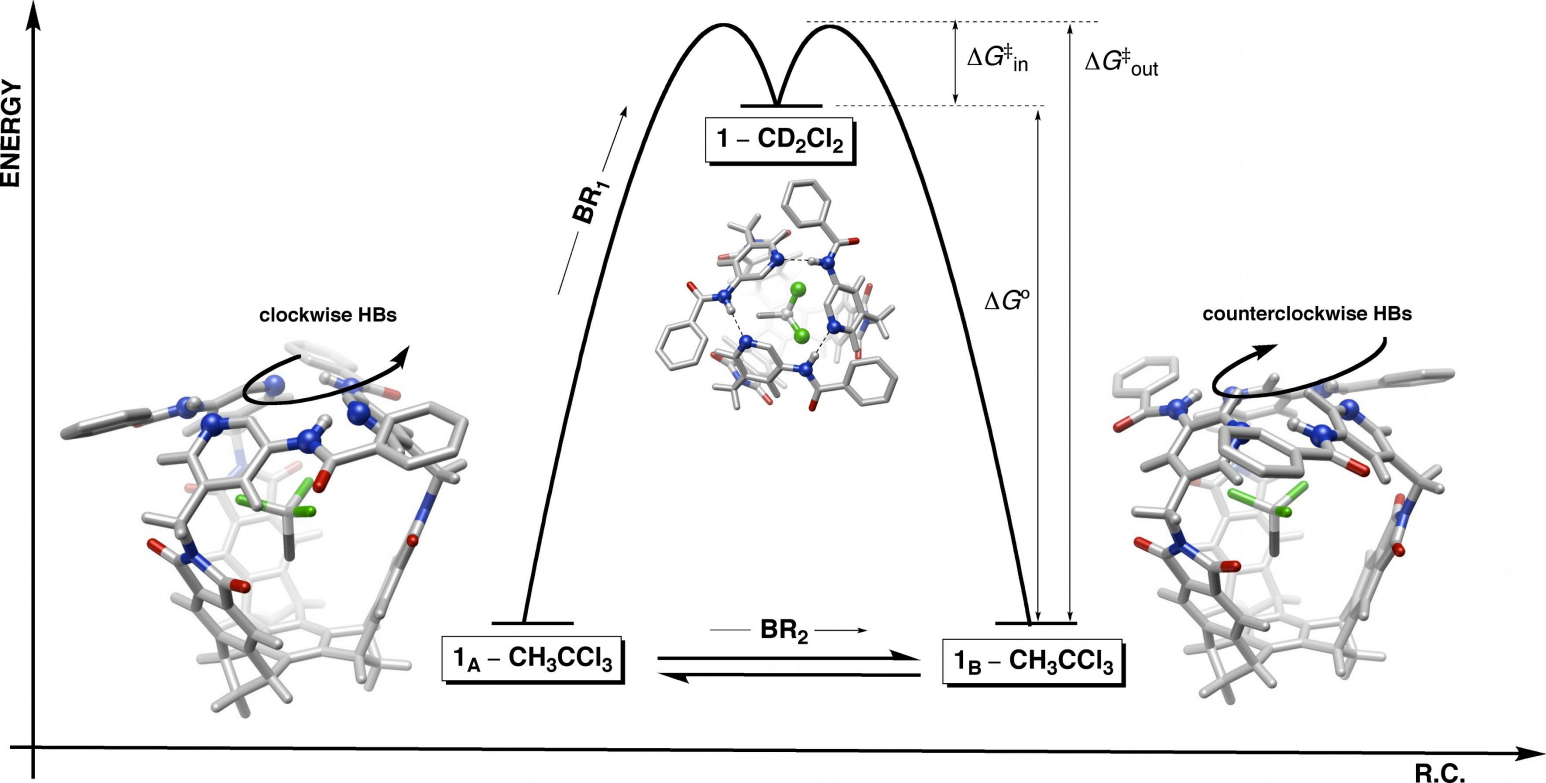
The departure of CH_3_CCl_3_ from **1**_A_–CH_3_CCl_3_ gives rise to the less stable **1**–CD_2_Cl_2_, which upon entrapment of another CH_3_CCl_3_ gives either **1**_A_–CH_3_CCl_3_ or **1**_B_–CH_3_CCl_3_. The **1**_A/B_–CH_3_CCl_3_ interconversion occurs with CH_3_CCl_3_ departing (BR_1_ mechanism) or remaining (BR_2_ mechanism) in the cavity.

Is there a relationship between the aromatic gates sweeping the space and guests trafficking to and from the basket [11]? That is to say, will the gates expel the entrapped guest each time that they alter their propeller-like orientation (Figure 8)? First, our kinetic measurements suggest that guest CH_3_CCl_3_ (**2**) enters basket **1** by substituting solvent (CD_2_Cl_2_) molecule(s), while exactly the opposite occurs during the dissociation (Figure 8). Given this exchange scenario, we deduce that **1_A_**–CH_3_CCl_3_ shall transform into **1**_B_–CH_3_CCl_3_ via intermediate **1**–CD_2_Cl_2_ (Figure 8). That is, the formation of **1**–CD_2_Cl_2_ from **1_A_**–CH_3_CCl_3_ is accompanied by either reorientation or reinstatement of the gates, and therefore, there is an equal likelihood that **1**–CD_2_Cl_2_ will yield **1_A_**–CH_3_CCl_3_ or **1_B_**–CH_3_CCl_3_ (Figure 8); this reasoning is also supported by the fact that the gates of the solvated basket revolve at a higher rate (Figure 7B). In accordance with such a racemization mechanism, we apply the statistical correction to the measured *k*_rac_ to obtain *k*_rac′_ (*k*_rac′_ = 2*k*_rac_ = 616 s^−1^, Figure 7B) [34]. This particular rate coefficient should more precisely describe the process of racemization.

One could describe the free energy characterizing the guest departure (Δ*G*^‡^_out_) as a linear combination of Δ*G*^‡^_rac′_ + Δ*G*° + Δ*G*^‡^_sterics_ representing (1) the opening of the gates (Δ*G*^‡^_rac′_), (2) the decomplexation of the guest (Δ*G*°), and (3) the “slippage” of the guest while exiting the open host (Δ*G*^‡^_sterics_) [8,11,21,35]. The encapsulation kinetics is first-order in guest CH_3_CCl_3_ suggesting that this species creates van der Waals strain (friction) during the in/out trafficking, thereby justifying the use of the Δ*G*^‡^_sterics_ term.

In addition, the decomplexation of CH_3_CCl_3_ follows a late transition state [14] whereby its affinity for populating the interior of the basket should decrease to a somewhat smaller value than described by Δ*G*°. Given the delicacy of the proposed partitioning, will the additivity of free energies and the relationship Δ*G*^‡^_rac′_ + Δ*G*° + Δ*G*^‡^_sterics_ ~ Δ*G*^‡^_out_ still hold?

When Δ*G*^‡^_rac′_ of 11.4 ± 0.1 kcal/mol (at 250.0 ± 0.1 K, Figure 7) is added to the intrinsic binding energy of CH_3_CCl_3_ (│Δ*G*°│ = 2.79 ± 0.09 kcal/mol at 250.0 ± 0.1 K, Figure 2), a value of 14.2 kcal/mol is obtained. Without even including Δ*G*^‡^_sterics_ (as a positive number), there is an apparent disagreement between the sum value (≥14.2 kcal/mol) and the experimentally determined Δ*G*^‡^_out_ = 13.4 ± 0.1 kcal/mol (from 2-D EXSY, *k*_out_ = 10 ± 1 s^−1^). Is there a missing factor needed in order to understand this phenomenon?

In reality, when the internal hydrogen bonds are broken and the gates open up the guest does not have to depart the basket cavity. That is to say, the gates should be able to revolve to allow the interconversion of **1_A_**–CH_3_CCl_3_ into **1**_B_–CH_3_CCl_3_ without even ejecting the guest. Accordingly, we hereby propose that the conversion of **1_A_**–CH_3_CCl_3_ into **1**_B_–CH_3_CCl_3_ (i.e., racemization) occurs by two routes, BR_1_ and BR_2_, one with (BR_1_) and another without (BR_2_) the concomitant guest exchange (Figure 8).

It follows that, during the departure of CH_3_CCl_3_, the measured racemization of **1** (Δ*G*_rac′_) includes energetic contributions from two pathways (Δ*G*^‡^_rac′_ = Δ*G*^‡^_BR1_ + Δ*G*^‡^_BR2_) of which only BR_1_ should be incorporated in the additivity assessment. It is therefore convenient to partition the energetic contribution of the two “competing” BR_1_ and BR_2_ routes to Δ*G*^‡^_rac′_ (Δ*G*^‡^_rac′_ = Δ*G*^‡^_BR1_ + Δ*G*^‡^_BR2_) to corroborate fully the role of the gates. However, this is a difficult task, but for guest molecules holding strongly onto the basket (more negative Δ*G*°) there should be a greater contribution from the RG_2_ pathway during the racemization.

**Figure 9:**
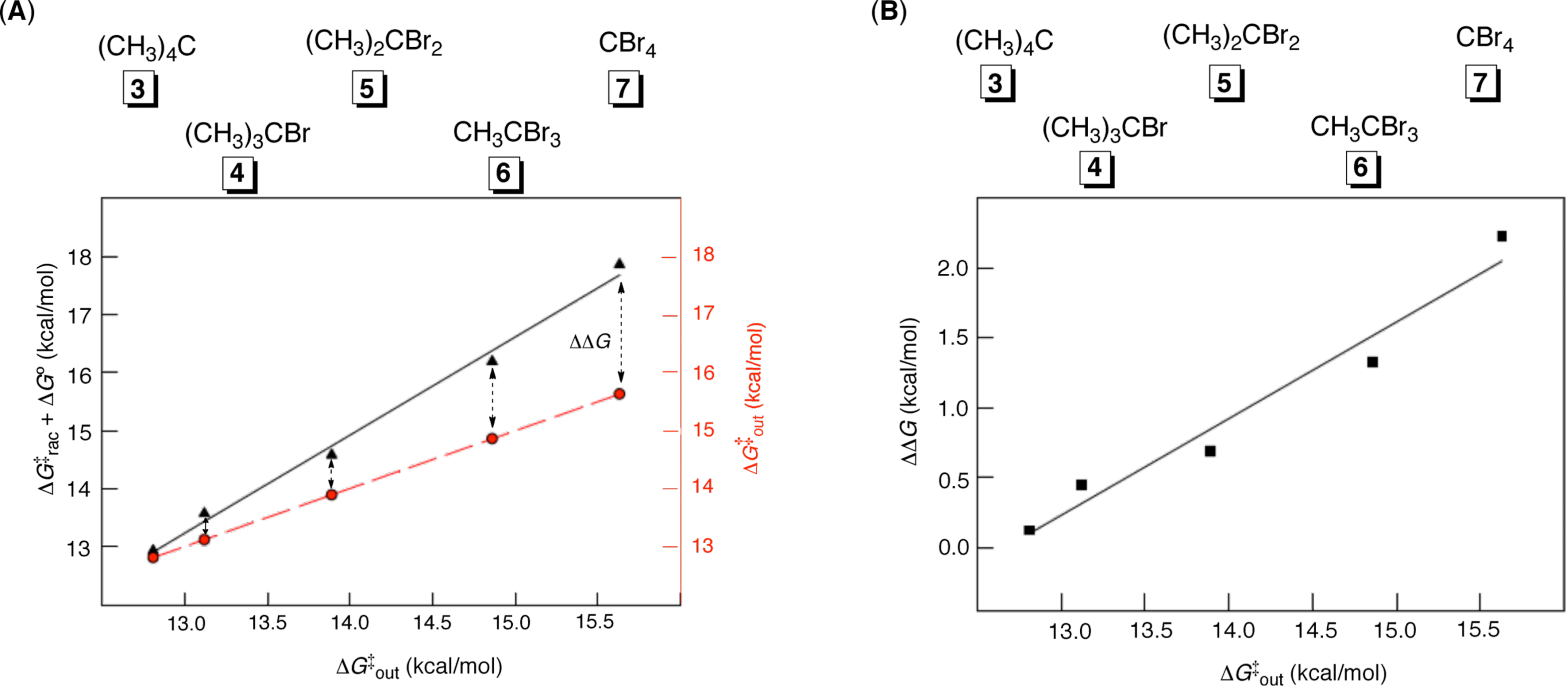
(A): Kinetic and thermodynamic parameters [13–14] characterizing the departure of isosteric guests **3**–**7** from the basket. (B): The computed ΔΔ*G* = (Δ*G*^‡^_rac′_ + Δ*G*°) − Δ*G*^‡^_out_ is apparently a linear function of Δ*G*^‡^_out_.

In one of our prior studies [13–14], we measured kinetic and thermodynamic parameters pertaining to the exchange of five isosteric (same-size) guests **3**–**7** to and from basket **1** (Figure 9). When Δ*G*^‡^_rac′_ + Δ*G*° is computed for each guest and the values plotted against Δ*G*^‡^_out_, a linear relationship appears (*R*^2^ = 0.99, Figure 9A). Note that Δ*G*^‡^_sterics_ is not included in this analysis as it is unknown; however, we anticipate that the value of the parameter should show minimal fluctuations for the series of isosteric guests **3**–**7**. Importantly, the greater the affinity of a particular guest for occupying the interior of the basket (Δ*G*°), the greater the deviation of the calculated Δ*G*^‡^_rac′_ + Δ*G*° (black line, Figure 9A) from the experimental Δ*G*^‡^_out_ (red line, Figure 9A). The variation of ΔΔ*G* = (Δ*G*^‡^_rac′_ + Δ*G*°) − Δ*G*^‡^_out_ with intrinsic binding energies Δ*G*° of **3**–**7** is shown in Figure 9B. The trend is evident, supporting the notion that for guests having greater propensity to occupy the basket (Δ*G*°) the BR_2_ pathway is more greatly involved in the **1_A_**–CH_3_CCl_3_/**1_B_**–CH_3_CCl_3_ racemization. As already discussed, the BR_2_ pathway contributes to the measured Δ*G*^‡^_rac′_, yet it is not involved in the exchange of guests.

## Conclusion

Describing mechanisms by which dynamic hosts entrap/release guests is a challenging task necessitating experimental and computational scrutiny. Notably, one can use NMR spectroscopic methods for understanding the equilibrium kinetics characterizing the rate law of molecular encapsulation processes. Our study, accordingly, describes the rate law characterizing the encapsulation of guest CH_3_CCl_3_ by the gated basket **1.** Importantly, the entrapment reaction is first-order in each compound, while the complex dissociation is zeroth-order in guest CH_3_CCl_3_. Furthermore, examination of the additivity of free energies corresponding to different molecular events can assist in the understanding of the operation of gated hosts and, in particular, can help to reveal the explicit role of the gates. On the basis of these results, we deduced that the synchronicity in the revolving motion of the gates and in/out trafficking of guests is a function of the affinity of the guest for occupying the gated basket. The greater the affinity, the less effective the gates are in “sweeping” the guest as the gates undergo their revolving motion. This result is important for exploring the utility of gating for controlling the outcome of chemical reactions occurring in confined space but also for the understanding of the effective conversion of energy at the molecular level and the preparation of molecular machines [36–37].

## Experimental

**Procedure for 2-D EXSY experiments** [25]: A solution of basket **1** and guest **2** in CD_2_Cl_2_ (J. Young NMR tube) was cooled to 250.0 ± 0.1 K inside the NMR probe and allowed to equilibrate for 1.0 h. A series of gradient NOESY experiments was run with a relaxation delay of 5 × *T*_1_ and mixing times (τ_m_) of 0 ms and three others ranging from 40 ms to 250 ms, such that the cross-peaks were clearly resolved; the spin–lattice relaxation time (*T*_1_ = 3.30 s) for the free guest was determined by performing a standard inversion-recovery pulse sequence with a relaxation delay (τ_d_) of at least 5 × *T*_1_. Each of the 128 F_1_ increments represented the accumulation of at least two scans. The corresponding integrals were determined by using MNova software from Mestrelab Research, after phase and baseline corrections in both dimensions. The magnetization exchange rate constants (k*_in_ and k*_out_) were, at each mixing time τ_m_, calculated by using the EXSYCalc program (Mestrelab Research). The mean values of k*_in_ and k*_out_ are reported with the standard deviation as an experimental error.

**Procedure for ^1^H-selective inversion-transfer experiments** [27]: A solution of basket **1** and guest **2** in CD_2_Cl_2_ (J. Young NMR tube) was cooled to 250.0 ± 0.1 K inside the NMR probe and allowed to equilibrate for 1.0 h. The ^1^H spin–lattice relaxation time (*T*_1_ = 3.30 s) for the free guest was determined by a standard inversion-recovery pulse sequence with a relaxation delay (τ_d_) of at least 5 × *T*_1_. By using a selective 1-D inversion-recovery pulse sequence [180° x (selective) – τ – 90° x (nonselective) – τ_d_], 32 transients were obtained for each variable delay time (τ) with a relaxation delay (τ_d_) of at least 5 × *T*_1_. The absolute integrals corresponding to encapsulated and free guest molecules were, at each mixing time, determined by using TopSpin software from Bruker, and the resulting data was fitted by using the two-site exchange equations described by Led et al. [27] to obtain magnetization exchange rate constants k*_in_ and k*_out_.

## Supporting Information

Supporting Information contains details of the computational studies.

File 1 Details of the computational studies.
