# Supplementary material for: On the mechanism of action of gated molecular baskets: The synchronicity of the revolving motion of gates and in/out trafficking of guests
Source: Beilstein J Org Chem. 2012 Jan 16;8:90–9. doi: 10.3762/bjoc.8.9 (PMC3302102; doi:10.3762/bjoc.8.9)
Supplement: File 1 — Details of the computational studies. [file Beilstein_J_Org_Chem-08-90-s001.pdf]

Supporting Information

for

On the mechanism of action of gated molecular baskets: The synchronicity of the revolving motion of gates and in/out trafficking of guests

Keith Hermann, Stephen Rieth, Hashem A. Taha, Bao-Yu Wang, Christopher M. Hadad and Jovica D. Badjić*

Address: Department of Chemistry, The Ohio State University, 100 West 18th Avenue, Columbus OH, 43210, USA

Email: Jovica D. Badjić - Badjić@chemistry.ohio-state.edu

* Corresponding author

Details of the computational studies

All molecular dynamics (MD) and steered molecular dynamics (SMD) simulations were carried out as described previously [1] by using the Amber 10.0 suite of programs [2]. The Amber ff03 force field [3] and the GAFF force field [4] were employed for the molecular basket and the guest during these simulations. Partial atomic charges for the guest were computed with Gaussian 09 [5] by using the CHELPG method [6] at the B3LYP/6-31G(d,p) level of theory. The complex was surrounded by a cubic box of chloroform with a margin of 10.0 Å along each dimension. Electrostatic interactions were calculated by using the particle-mesh Ewald (PME) algorithm [7] with a cutoff of 10 Å applied to the Lennard–Jones interactions. Bonds involving hydrogen were constrained by utilizing the SHAKE algorithm [8], and periodic boundary conditions were employed in all calculations. Prior to the MD simulation, 1000 steps of energy minimization were performed for the solvent with fixed basket and followed by another 2500 steps of energy minimization for the whole system. Then the entire model system was heated up from 0 to 300 K gradually over 20 ps with the molecular basket restrained by a weak harmonic potential. Subsequently, a 10 ns MD simulation was carried out at 1 atm and 300 K under the NPT ensemble with the Langevin temperature scheme. The final equilibrium state of the MD simulation was used as the starting point for the subsequent steered molecular dynamics (SMD) studies [9]. SMD simulations were performed under NPT conditions for 2 ns. External restraints were employed to pull the guest out of the molecular basket by using multiple pathways (see below).

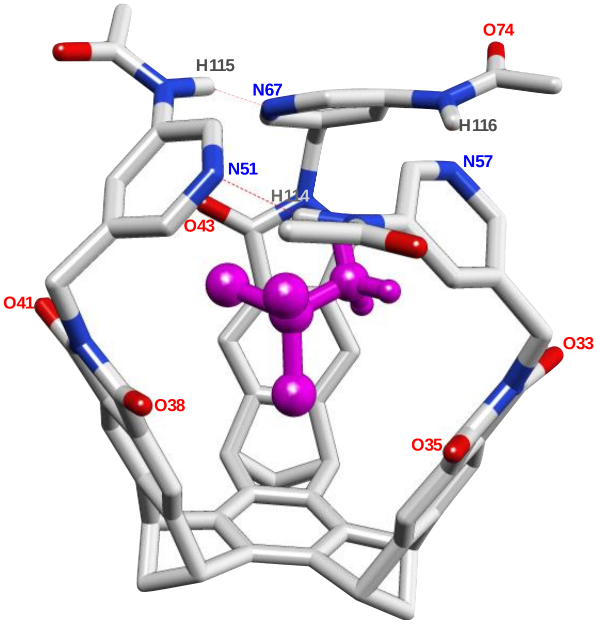

Figure S1: Definition of atom numbering in basket.

The N-H···N hydrogen bond distances along the top opening of the basket were monitored throughout the 10 ns simulations of the molecular basket with CH_3CCl_3 inside; all three H-bonds were found to be constant (~2 Å, Figure S2) with intermittent fluctuations (up to 3.5 Å).

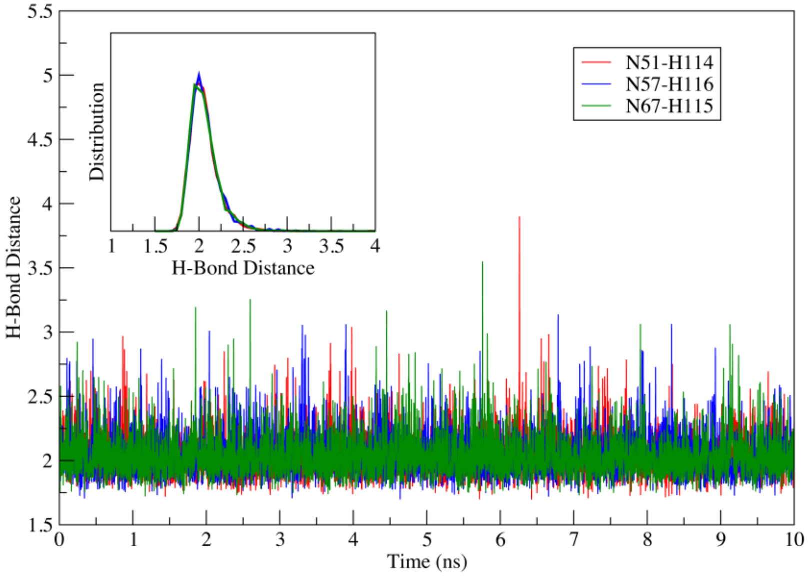

Figure S2: N-H···N distances as a function of time in the 10 ns MD simulation of the basket containing CH_3CCl_3. The inset shows the distribution of H-bond distances.

The widths of the side apertures also remain constant at ~6.3 Å throughout the simulations with ~1 Å fluctuations (Figure S3).

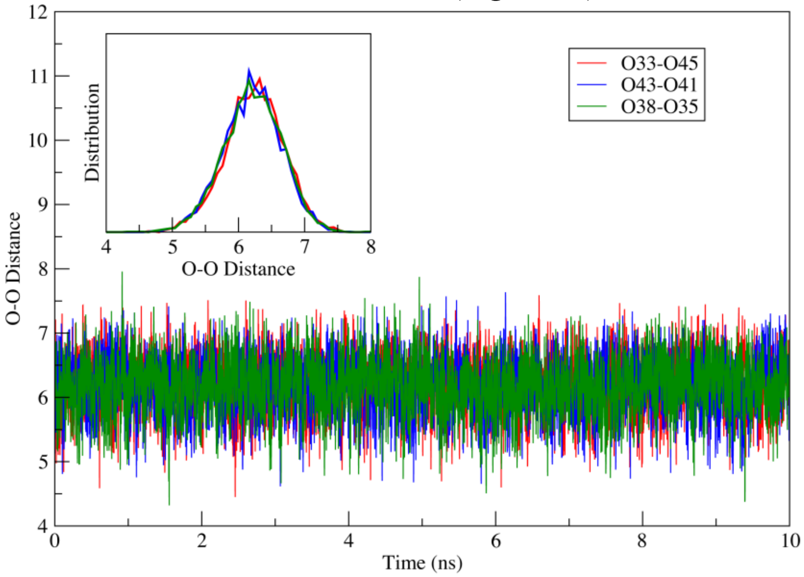

Figure S3: Side aperture widths (i.e., O–O distances) as a function of time in the 10 ns MD simulation of the basket containing CH_3CCl_3. The inset shows the distribution of distances.

The guest CH_3CCl_3 was pulled from the inside of the basket by using several different pathways (see Scheme S1).

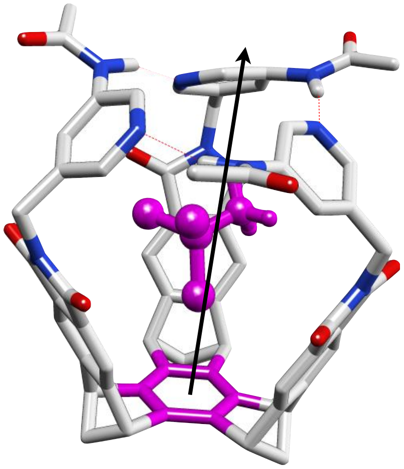

BASE Pull

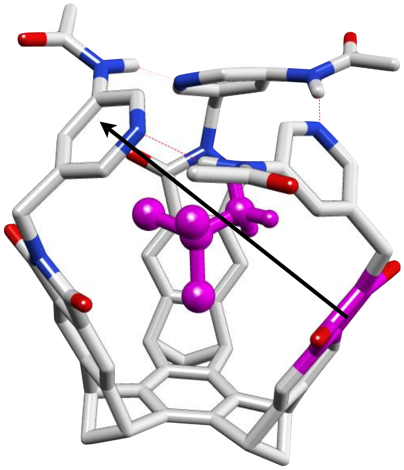

N36ARM Pull

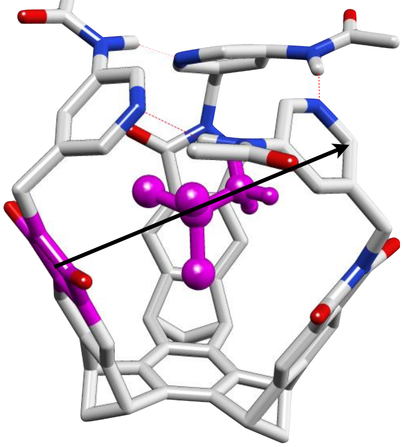

N39ARM Pull

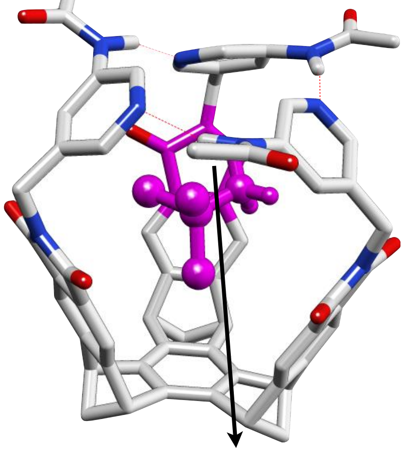

N46ARM Pull

Scheme S1: Different pathways for pulling the guest CH_3CCl_3 molecule from the basket. The pulling direction is defined by the indicated vector.

During each pull, the N–H···N H-bond distances and side aperture O-O distances were monitored. In addition, complete H-bond analyses were carried out on all resulting trajectories. In all cases, at least two N–H···N H-bonds had to be broken upon departure of the guest, and the respective side apertures expand (see figures below). In some cases, the initial N–H···N bonds are reformed after the guest leaves the basket. In others, formation of new H-bonds is observed between N–H and the C=O groups.

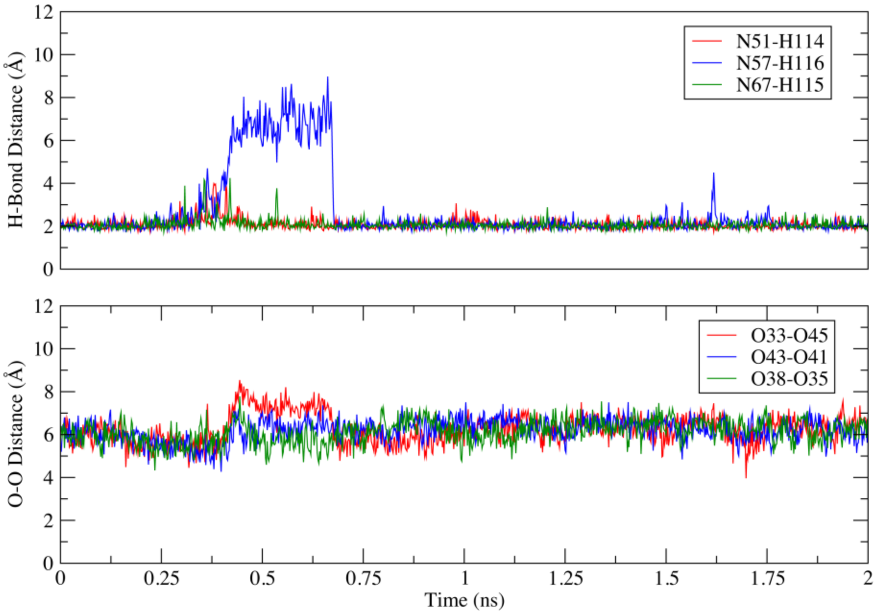

Figure S4: Variation in H-bonds (A) and O–O distance (B) of the molecular basket during the SMD simulations (BASE Pull).

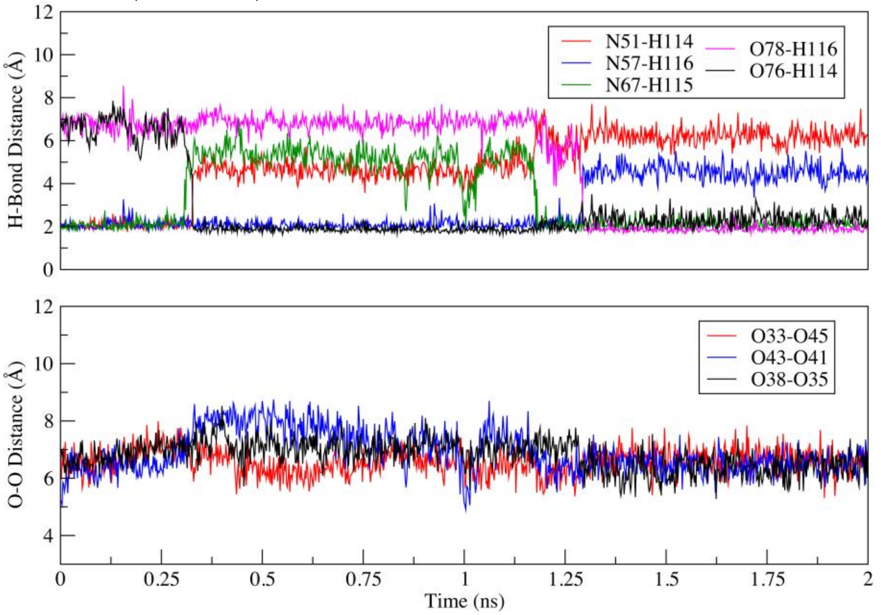

Figure S5: Variation in H-bonds (A) and O–O distance (B) of the molecular basket during the SMD simulations (N36ARM Pull).

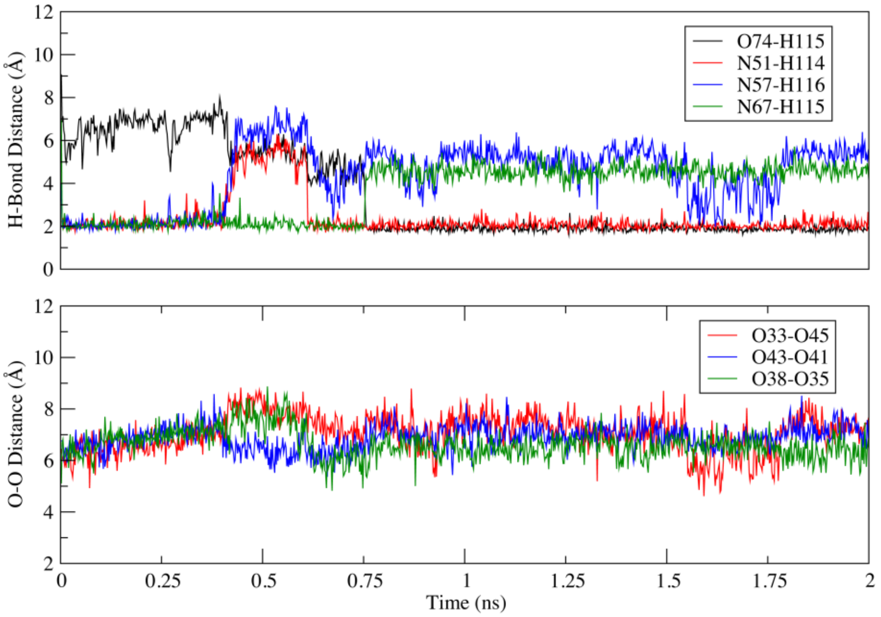

Figure S6: Variation in H-bonds (A) and O–O distance (B) of the molecular basket during the SMD simulations (N39ARM Pull).

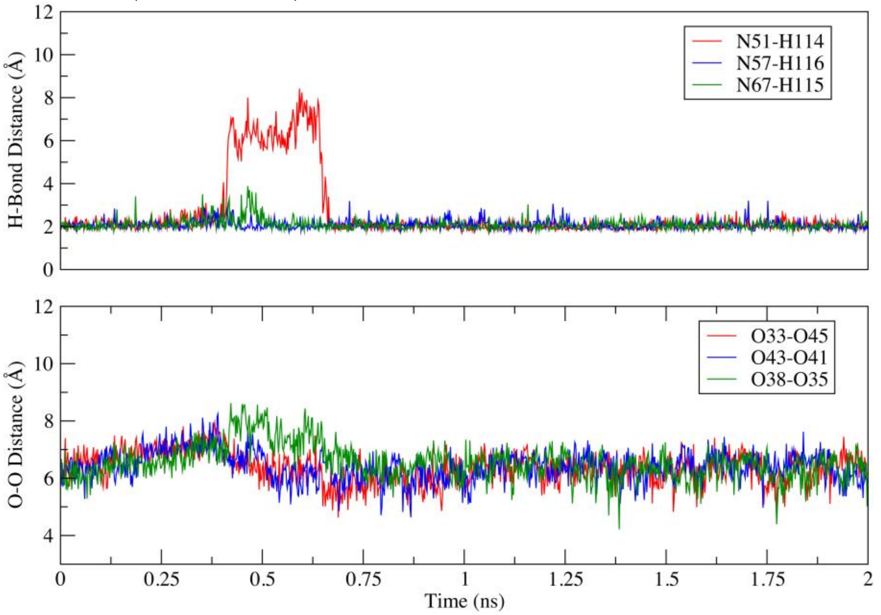

Figure S7: Variation in H-bonds (A) and O–O distance (B) of the molecular basket during the SMD simulations (N46ARM Pull).

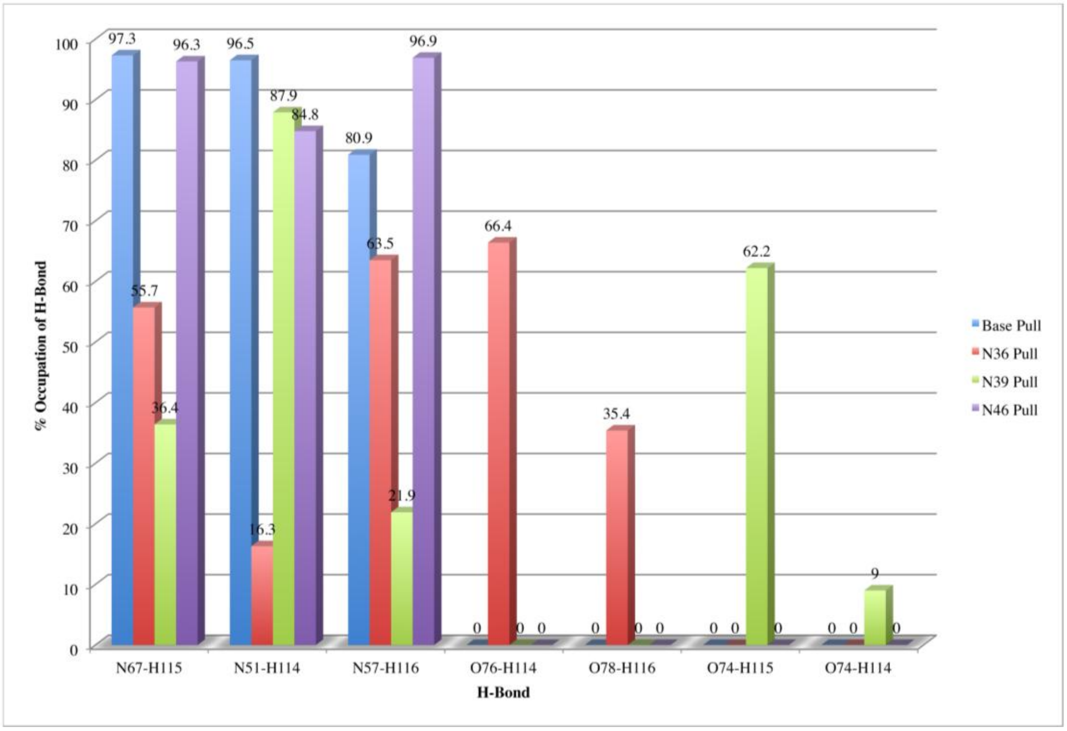

Figure S8: H-bond analysis of the molecular basket during the SMD simulations.

References

1. Rieth, S.; Bao, X.; Wang, B.-Y.; Hadad, C. M.; Badjić, J. D. *J. Am. Chem. Soc.* **2010,** *132,* 773. doi:10.1021/ja908436c
2. AMBER 10; University of California: San Francisco, 2008.
3. Duan, Y.; Wu, C.; Chowdhury, S.; Lee, M. C.; Xiong, G.; Zhang, W.; Yang, R.; Cieplak, P.; Luo, R.; Lee, T.; Caldwell, J.; Wang, J.; Kollman, P. *J. Comput. Chem.* **2003,** *24,* 1999. doi:10.1002/jcc.10349
4. Wang, J.; Wolf, R. M.; Caldwell, J. W.; Kollman, P. A.; Case, D. A. *J. Comput. Chem.* **2004,** *25,* 1157. doi:10.1002/jcc.20035
5. Gaussian 09, Revision A.01; Gaussian, Inc.: Wallingford, CT, 2009.
6. Breneman, C. M.; Wiberg, K. B. *J. Comput. Chem.* **1990,** *11,* 361. doi:10.1002/jcc.540110311
7. Essmann, U.; Perera, L.; Berkowitz, M. L.; Darden, T.; Lee, H.; Pedersen, L. G. *J. Chem. Phys.* **1995,** *103,* 8577. doi: 10.1063/1.470117

8. Ryckaert, J. P.; Ciccotti, G.; Berendsen, H. J. C. *J. Comput. Phys.* **1977,** *23,* 327. doi:10.1016/0021-9991(77)90098-5
9. Isralewitz, B.; Gao, M.; Schulten, K. *Curr. Opin. Struct. Biol.* **2001,** *11,* 224. doi:10.1016/S0959-440X(00)00194-9
